# Reconstitution of genome remodeling using *Xenopus* egg extracts

**DOI:** 10.3389/fcell.2026.1932329

**Published:** 2026-09-17

**Authors:** Katherine G. Lenz, Coral Y. Zhou

**Affiliations:** Department of Molecular Biosciences, University of Kansas, Lawrence, KS, United States

**Keywords:** chromatin assembly and remodeling, chromosome segregation, DNA repair, DNA replication, genome size, nuclear assembly, nuclear size, *Xenopus*

## Abstract

Changes in 3D genome organization drive critical cellular transitions including cell division and differentiation. Cytoplasmic extracts derived from eggs of the African clawed frog *Xenopus laevis* offer a powerful system for reconstituting genome remodeling events *in vitro* and dissecting the underlying biochemical mechanisms. Female *X. laevis* produce thousands of large eggs that can be readily lysed by centrifugation to yield egg cytoplasm—commonly referred to as egg extract. This extract contains the full complement of molecules required to support multiple rounds of chromatin assembly, nuclear formation, DNA replication and repair, and chromosome segregation. Addition of an exogenous DNA source triggers rapid, highly reproducible remodeling that can be monitored by fluorescence microscopy, gel electrophoresis, or genomics approaches. The biochemical tractability of this system has historically been instrumental in revealing fundamental mechanisms of chromatin assembly and remodeling. This review summarizes the major genome remodeling assays enabled by *Xenopus* egg extracts and highlights recent advances in the field. We also outline promising new directions for leveraging this system to unravel the molecular basis of speciation and polyploidy.

## Introduction

Chromatin is dynamically remodeled by a wide range of molecular machines to facilitate major cellular transitions such as cell division, cell signaling, and differentiation. Studying the molecular mechanisms of chromatin remodeling is critical for our fundamental understanding of how genomes are stored, propagated, and protected in order to specify cell states and promote organism health (Zhou et al., 2016; Zhang and Cao, 2021; Ito and Zaret, 2022). A major challenge in studying biochemical mechanisms of genome remodeling is that these processes require complex machinery, making reconstitution from pure components nearly impossible. Also, the genes involved are often essential, making genetic perturbations difficult.

Cytoplasmic extracts from the eggs of the African clawed frog *Xenopus* are made by crushing eggs in a centrifuge to separate the cytoplasm from lipids and yolk (Figure 1A). Egg extracts offer a unique approach to studying chromatin dynamics by reconstituting genome remodeling events *in vitro* (Lohka and Masui, 1983; 1984; Maresca and Heald, 2006; Edens and Levy, 2016; Rankin, 2018). Egg extracts are stockpiled with all of the molecules required to perform essential genome remodeling processes including nuclear assembly, chromatin assembly, DNA replication, DNA repair, and chromosome segregation. Addition of a genome source, such as a plasmid, DNA beads, or nuclei into egg extracts initiates rapid and controlled genome remodeling. To interrogate mechanisms of genome remodeling, extracts can be biochemically manipulated through immunodepletion and add-back experiments (Jenness et al., 2018b; Mevissen et al., 2023). Small molecules such as fluorescent probes or drugs can also be added to extracts at any concentration. Finally, extracts are naturally arrested in metaphase but can be easily transitioned into interphase with addition of calcium, allowing for investigation of both phases of the cell cycle (Maresca and Heald, 2006). Here we review how *Xenopus* egg extracts have been used to investigate different types of chromatin remodeling, with a focus on the mechanisms that have been revealed in recent years.

**FIGURE 1:**
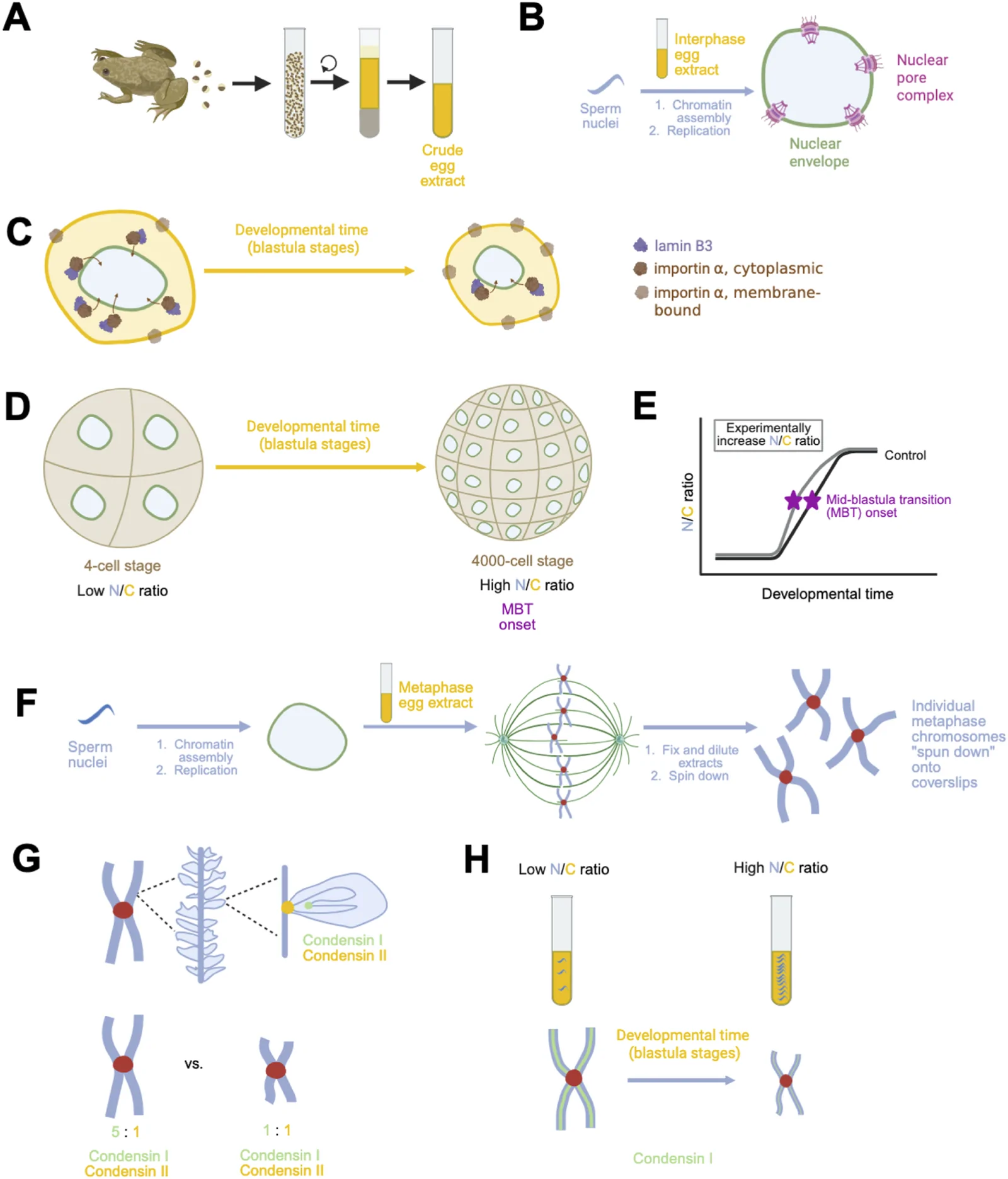
Reconstitution of nuclei and mitotic chromosomes using *Xenopus* egg extracts. **(A)** Female *Xenopus* lay thousands of eggs overnight. To make egg extracts, eggs are collected in a centrifuge tube and fractionated to separate the cytoplasm (middle golden layer) away from yolk, pigments and lipids. **(B)** When sperm nuclei are added to interphase egg extracts, maternal factors assemble chromatin and replicate the DNA, and full nuclear envelopes are assembled. **(C)** During the blastula stages of early development, cell size decreases due to rapid cell division events in the absence of cell growth. Nuclear size scales with cell size due to the partitioning of importin α toward the cell membrane as cell size decreases, resulting in less cytoplasmic importin α to import nuclear scaling factors such as lamin B3. **(D)** During the blastula stages, the nuclear-to-cytoplasmic (N/C) ratio increases due to increasing genome copies in a fixed amount of cytoplasm. After the embryo reaches ∼4000 cells, the mid-blastula transition (MBT) begins. During MBT, the cell cycle slows, 3D genome organization is established, and zygotic transcription begins. **(E)** Experimentally increasing N/C ratio results in early onset of MBT. **(F)** To reconstitute mitotic chromosomes, sperm nuclei are first added to interphase egg extracts. After the chromatin is assembled and DNA is replicated, fresh metaphase extracts are added to form mitotic chromosomes and the bipolar mitotic spindle. To isolate and image individual chromosomes, the extract is diluted with fixative and chromosomes are “spun down” onto coverslips. **(G)** The basic unit of the mitotic chromosome is the DNA loop, which is formed by condensin I and II. Condensin II forms large DNA loops, while condensin I forms smaller DNA loops nested within the condensin II loops (Gibcus et al., 2018). Depletion and add-back studies in *Xenopus* extracts showed that artificially changing the ratio of condensin I to condensin II changes the size of mitotic chromosomes (Shintomi and Hirano, 2011). **(H)** Artificially increasing N/C ratio in egg extracts to simulate what occurs naturally during early embryogenesis results in decreased condensin I loading and shorter mitotic chromosomes, demonstrating that N/C ratio is a developmental cue that regulates chromosome size in the embryo (Zhou et al., 2023).

## Reconstituting nuclear assembly

To reconstitute nuclear assembly, purified sperm nuclei are added to interphase egg extracts. Within an hour, the DNA is packaged into chromatin, replicated by maternal factors, and full nuclear envelopes capable of nuclear trafficking are formed (Maresca and Heald, 2006; Holzer and Antonin, 2022; Mishra and Levy, 2022; Ikeda et al., 2026) (Figure 1B). Early studies using egg extracts revealed nucleoplasmin as a key maternal factor that removes sperm-specific factors from the paternal genome, allowing for loading of maternal histones after fertilization (Ohsumi and Katagiri, 1991; Philpott et al., 1991; Philpott and Leno, 1992; Leno et al., 1996). Recently, these types of assays have been critical for understanding the molecular mechanisms governing nuclear size and nuclear-to-cytoplasmic (N/C) partitioning.

Previously, it was shown that differential rates of nuclear import tune nuclear size across species and during development. In particular, species with larger nuclei contain higher levels of importin α, which binds and imports cargo with nuclear localization sequences (NLS) into the nucleus including lamin B3, a major component of the nuclear envelope (Levy and Heald, 2010). During early development, cell size decreases exponentially (Levy and Heald, 2012). Importin α scales nuclear size to cell size by partitioning to the cell membrane as cell size decreases, thus becoming less available to import lamin B3 (Figure 1C). More recently, additional factors that regulate nuclear size during development have been identified including the histone chaperone Npm2 (Chen et al., 2019), and the conventional protein kinase C (cPKC) (Edens and Levy, 2014). DNA amount also contributes to nuclear size. Decreasing DNA content results in slower nuclear growth and lower nuclear size at steady state (Heijo et al., 2020). In some cases, nuclear growth depends solely on DNA amount and is completely uncoupled from nuclear import (Chen et al., 2023). Increasing the compaction of the DNA resulted in slower nuclear growth, suggesting that DNA structure, and not just amount, can regulate nuclear size. For example, treating the extracts with drugs that increase the amount of heterochromatin increased nuclear size without changing nuclear import rates (Chen et al., 2023). Together these studies highlight how nuclear size is regulated by many context-dependent factors including nuclear import, DNA amount, DNA condensation, as well as developmentally regulated factors.

The nuclear-to-cytoplasmic (N/C) volume ratio is a tightly regulated parameter that has important biological functions, but regulation of the N/C ratio is poorly understood (Balachandra et al., 2022). During the blastula stages of early development, the N/C ratio rises exponentially due to rapid cell cycle events in the absence of cell growth (Balachandra et al., 2022). The N/C ratio is thought to regulate the timing of the mid-blastula transition (MBT), when zygotic transcription begins and the cell cycle slows down (Amodeo et al., 2015; Jukam et al., 2017; 2021) (Figure 1D). Experimentally increasing N/C ratio prematurely activates MBT (Jevtić and Levy, 2015), while decreasing nuclear size delays zygotic gene expression and cell cycle slow down (Jevtić and Levy, 2017) (Figure 1E). In another study, increasing nuclear size correlated with increased cell cycle duration across extracts and nuclei from different species of *Xenopus*, suggesting that nuclear volume can directly regulate changes in cell cycle duration during MBT (Piñeros et al., 2025). Finally, recent studies using optical diffraction tomography to measure density in egg extracts revealed that while N/C volume ratio changes drastically during development, an N/C density ratio of approximately 0.8 is conserved throughout development and across vastly different eukaryotes (Biswas et al., 2025). Experimental deviations from this conserved ratio resulted in increased cell senescence, suggesting that N/C density, in addition to volume, is an additional biophysical parameter that must be tightly and universally regulated in order to promote normal cell physiology. Future work will reveal the molecular basis of this regulation, and how conserved those mechanisms are across species.

## Reconstituting mitotic chromosomes

During mitosis, duplicated chromosomes that are normally entangled during interphase must form individual, condensed, rod-shaped structures that align at the center of the mitotic spindle (Kinoshita and Hirano, 2017; Batty and Gerlich, 2019). The factors required for chromosome segregation are highly dynamic, and the window of time in which division occurs is very short, making it difficult to isolate the roles of individual factors in this process (Maddox et al., 2006). Also, individual mitotic chromosomes are very difficult to discern in a living cell, making it nearly impossible to measure their dimensions. *Xenopus* egg extracts are naturally and indefinitely arrested in metaphase, enabling prolonged imaging and dissection of molecular mechanisms (Shintomi and Hirano, 2016; Silva and Rankin, 2018). In a typical experiment, sperm nuclei are added to interphase egg extracts to allow for replication. After one hour, metaphase egg extracts are added, resulting in the formation mitotic chromosomes by condensin complexes (Figure 1F). This process can either be visualized live, or extracts containing chromosomes can be diluted, fixed, and “spun down” onto coverslips to visualize individual chromosomes (Silva and Rankin, 2018) (Figure 1F). Importantly, this assay was used to discover the six components that are sufficient to create mitotic chromosomes, which include histones, histone chaperones, topoisomerase II, and condensin I (Shintomi et al., 2015). More recently, variations of this assay have been used to investigate a major open question in the field–how do molecules that operate on the nanometer scale come together to create micron-long mitotic chromosomes of a specific shape and size?

Condensin I and II are ATP-dependent motors that create and maintain a series of DNA loops that emanate from the central scaffold of each mitotic chromosome (Earnshaw and Laemmli, 1983; Hirano, 2016; Ganji et al., 2018; Gibcus et al., 2018). Estimated loop sizes range from 60–400 kb during prometaphase, with smaller loops being formed by condensin I and nested within larger loops formed by condensin II (Gibcus et al., 2018) (Figure 1G). By depleting condensins from *Xenopus* egg extracts and adding back specific amounts of condensin I or II, one group showed that the ratio between the two types of condensins determine chromosome morphology (Shintomi and Hirano, 2011) (Figure 1G). Understanding what regulates condensin I loading has been a major question in the field. Surprisingly, condensin I can form mitotic chromosomes in the absence of nucleosomes (Shintomi et al., 2017). However, linker histone H1.8 inhibits condensin I binding, as depletion of H1.8 results in overloading of condensin I and hypercompaction of the chromosome (Choppakatla et al., 2021). In another study, increasing N/C ratio by increasing the amount of sperm nuclei added to egg extracts resulted in shorter mitotic chromosomes and less condensin I recruitment (Zhou et al., 2023) (Figure 1H). These results offer a mechanistic explanation for the gradual increase in mitotic chromosome compaction observed during the early blastula stages, a phenomenon termed mitotic chromosome scaling. While the biological significance of chromosome scaling is yet to be determined, this mechanism is the first of its kind for showcasing how mitotic chromosome shape can be naturally tuned during the lifetime of an animal.

How do cells keep condensin I activity off until they enter mitosis? One group concluded that condensin I is negatively regulated by the N-terminal tail of its kleisin subunit (Tane et al., 2022). Another group depleted condensin I and reintroduced recombinant complexes at varying concentrations to show that when the local concentration of condensins is high, two or more condensins can cooperatively drive mitotic chromosome formation (Kinoshita et al., 2022). More recently, this group has examined how chromatin arrangement affects overall mitotic chromosome shape. Because the typical *Xenopus* sperm chromatin is nucleosome-poor, they developed a modified egg extract assay using frog red blood cell nuclei, which carry dense, regularly spaced nucleosomes (Shintomi et al., 2026). In this system, proteins such as H1.8, the histone chaperone Nap1, and the ATP-dependent chromatin remodeler ISWI guide nucleosome spacing and chromosome shaping (Shintomi and Hirano, 2021; Shintomi et al., 2026) A separate group showed that TFIIH, a general transcription factor, also plays a role in mitotic chromosome formation, further suggesting that while nucleosomes are not required for mitotic chromosome formation, the nucleosome landscape is still important for regulating condensin activity (Haase et al., 2022).

## Reconstituting DNA replication and repair

Maintaining a stable genome requires faithful replication of the genome as well as repair of DNA that has accumulated mutations or lesions. Malfunctions in genome maintenance can cause a wide range of deleterious consequences including cancer, premature aging, and developmental defects (O’Driscoll, 2012; Ribezzo et al., 2016). Egg extracts provide a powerful way of interrogating the biochemical mechanisms that perform genome maintenance. Not only do extracts contain all of the molecules that replicate and repair DNA, unnecessary components of the egg cytoplasm such as membranes can be removed by centrifugation to produce a highly concentrated, potent extract that efficiently replicates and repairs DNA termed high speed supernatant (HSS) (Maresca and Heald, 2006) (Figure 2A). Nucleoplasmic extract (NPE) concentrates nuclear factors even further. To make NPE, sperm nuclei are first replicated in interphase egg extract (Hoogenboom et al., 2017). Next, replicated nuclei are isolated and fractionated to collect only the nucleoplasm (Figure 2A). In a typical assay, a carefully designed plasmid DNA is added to either HSS and/or NPE to initiate a single full round of replication and/or repair (Lebofsky et al., 2009; Hoogenboom et al., 2017) (Figure 2B). The entire process can be easily tracked over time by separating substrates, intermediates, and products on a native gel, or by introducing fluorescent probes onto the DNA and performing live imaging (Lebofsky et al., 2009). By engineering DNA substrates to recapitulate different types of replication and repair intermediates, researchers have revealed important new mechanisms for how replisomes and DNA repair complexes perform their biochemical activities both during normal conditions and under stress (Hoogenboom et al., 2017).

**FIGURE 2:**
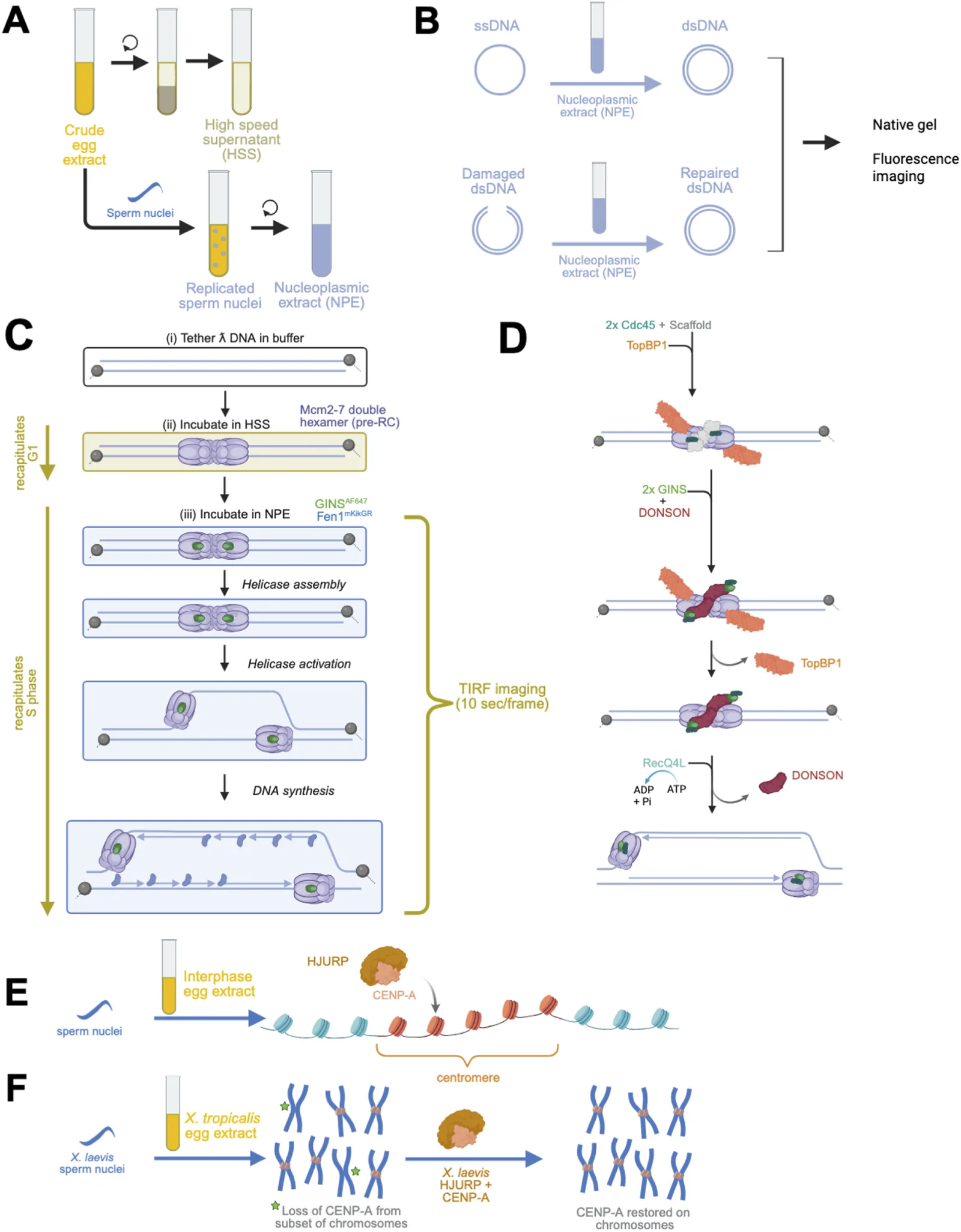
Reconstitution of DNA replication, repair, and centromere assembly using *Xenopus* egg extracts. **(A)** To make more potent versions of egg extract that are capable of entire rounds of DNA replication and repair on diverse DNA substrates, crude extracts are fractionated further to remove membranes, forming High Speed Supernatant (HSS). Alternatively, sperm nuclei are added to crude egg extracts to form nuclei, and the extract is fractionated further to collect only the nucleoplasm, forming Nucleoplasmic Extracts (NPE). **(B)** NPE can be used to fully replicate a single-stranded plasmid DNA (ssDNA) or repair damaged double-stranded DNA (dsDNA). Replication and repair reactions can be monitored by native gel or imaging. **(C)** In a single-molecule replication assay, λ DNA is tethered in a flow cell and placed in HSS to load the replicative helicase Mcm2-7. Next the DNA is transferred into NPE containing fluorescently labeled proteins to track movement of the helicase (GINS^AF647^), binding of different initiation factors, and newly synthesized DNA (Fen1^mKikGR^) using TIRF microscopy (Terui et al., 2024). **(D)** Model for how replication is initiated. First, two copies of Cdc45 and TopBP1 are recruited to Mcm2-7. Next, two copies of GINS and DONSON are recruited. Next, TopBP1 leaves the replication complex, and RecQ4L is recruited. The ATPase activity of RecQ4L is required to evict DONSON and allow replication to begin. **(E)** When sperm nuclei are added to interphase egg extracts, the DNA is fully assembled into chromatin. CENP-A, the centromeric histone that replaces canonical H3, is assembled onto centromeric DNA by its chaperone HJURP. **(F)** *Xenopus laevis* sperm is added to *X. tropicalis* egg extract to mimic an interspecies mating event. A subset of the mitotic chromosomes formed lack CENP-A, but this is rescued when supplementing the extract with *Xenopus laevis* HJURP and CENP-A (Kitaoka et al., 2022).

Understanding how origins are licensed and how replisomes engage in bidirectional movement are major questions in the replication field (Stillman et al., 2025). Using single-molecule imaging in extracts systematically depleted of different initiation factors, the precise order of recruitment of initiation factors was revealed for the first time (Berger and Chistol, 2023; Terui et al., 2024) (Figure 2C). First, TopBP1 is recruited to the two Mcm2-7 helicases at the initiation site, followed by dimers of the initiation factors Cdc45 and GINS, as well as DONSON (Terui et al., 2024). Finally, RecQ4L temporarily joins the complex to dissociate DONSON and uses its ATPase activity to initiate replication (Terui et al., 2024) (Figure 2D). After initiation, replisomes often encounter roadblocks in the form of nicked, damaged or single-stranded DNA (ssDNA), or other replisomes. How replisomes navigate these challenges has been unclear. Using egg extracts, we now understand that replisomes can completely disassemble or reverse direction, which may require re-initiation of completely new replisomes (Low et al., 2020; Vrtis et al., 2021; Kavlashvili et al., 2023). In summary, experiments using *Xenopus* egg extracts have revealed detailed molecular mechanisms for how genome stability is achieved under both normal and stressful conditions.

Under stress, replisomes may recruit additional factors to complete replication. For example, under topological stress, recruitment of a helicase RTEL1 and replication protein MCM10 to stalled replication forks facilitates termination (Campos et al., 2023; Kavlashvili et al., 2023). When replisomes encounter DNA-protein crosslinks (DPCs), the FANCJ helicase binds and unfolds the protein adduct, allowing the crosslink to be cleaved and resolved (Yaneva et al., 2023). DNA interstrand crosslinks (ICL) are formed by chemotherapeutics such as cisplatin, and present a major challenge to replisomes. (Rycenga and Long, 2018). When two replisomes converge on an ICL, the E3 ubiquitin ligase TRAIP ubiquitylates the replicative helicase, which recruits factors to either cleave the crosslink or to disassemble the replisome (Wu et al., 2019; Kochenova et al., 2025). Acetylaldehyde, a metabolite of alcohol consumption, also generates ICLs (Blouin and Saini, 2024). These damaged sites are repaired by a different pathway, using an error-prone mechanism that does not require DNA excision (Hodskinson et al., 2020).

## Reconstituting chromatin assembly

Faithful assembly of chromatin is critical for maintaining genome integrity and cell identity. In a classic assay for chromatin assembly, single-stranded plasmid DNA is added to interphase HSS, and within an hour the DNA is assembled into chromatin (Almouzni and Méchali, 1988; Almouzni et al., 1990). Assembly efficiency is measured by monitoring supercoiling of the plasmid on a native gel (Ray-Gallet and Almouzni, 2003). Combined with targeted immunodepletion and other biochemical methods, these types of experiments revealed two separate pathways that drive chromatin assembly–one that is replication-dependent, relying primarily on the histone chaperone CAF1, and a second replication-independent pathway that uses an alternative chaperone HIRA (Ray-Gallet et al., 2002; Ray-Gallet and Almouzni, 2003; Karagyozova et al., 2025).

A variation of this classic experiment is to pre-assemble nucleosomes, attach them to magnetic beads and use them as bait in pull-down experiments in egg extracts to discover new factors that bind to different types of chromatin. This approach was used to identify a unique ATP-dependent chromatin remodeling complex containing HELLS and CDC7 with *bona fide* nucleosome sliding activity (Jenness et al., 2018a; Wassing et al., 2024). Recently, one group used single particle cryo-EM to examine the structural differences of sperm chromatin assembled in interphase versus metaphase egg extracts at the level of single nucleosomes. They found that most nucleosomes, regardless of cell cycle stage, adopted the canonical left-handed octameric structure. Among the biggest variations they observed was in the position of linker histone H1.8, which bound closer to the dyad of the nucleosome in metaphase, resulting in a more closed conformation of linker DNA that could help with the compaction of chromosomes during mitosis (Arimura et al., 2021). The authors also created a novel method for enriching the pool of nucleosomes for a specific factor of interest using immunoprecipitation, called Magnetic Isolation and Concentration (MagIC)-cryo-EM. This new technique allows for structural analysis of low-abundant protein complexes with conformations that would otherwise be lost during class averaging (Arimura et al., 2025).

A specialized form of chromatin assembly is the formation of centromeres, the distinct region on the chromosome where chromosome segregation during mitosis takes place (McKinley and Cheeseman, 2016). Centromeres are defined epigenetically by the histone H3 variant CENP-A, which recruits a many additional factors to construct a centromere (McKinley and Cheeseman, 2016). Despite recent advances in characterizing centromeric DNA (Altemose et al., 2022), how centromeres are built is still unclear. Since centromeres are too complex to reconstitute from purified components and are essential for cell viability, a powerful approach is to study them in egg extracts (French and Straight, 2017). Adding purified sperm nuclei to egg extracts results in deposition of CENP-A (French and Straight, 2017) (Figure 2E). To date, this is the only cell-free system that assembles functional centromeres *de novo* and retains epigenetic inheritance of centromeres across the cell cycle (Desai et al., 1997; Guse et al., 2011; 2012). Experiments in egg extracts have revealed several key molecular players in centromere assembly including the CENP-A specific chaperone HJURP (Bernad et al., 2011), the Mis18 complex which primes chromatin for CENP-A loading during metaphase (French et al., 2017; Servin et al., 2023), and Cohesin (Haase et al., 2025).

Another key feature of centromeres is that the underlying DNA and associated machinery evolve rapidly, which can result in a reproductive barrier between even closely related species (Brown and O’Neill, 2010). For example, when *Xenopus laevis* sperm is used to fertilize *Xenopus tropicalis* eggs, the resulting hybrid embryo dies during gastrulation (Gibeaux et al., 2018). However, the reverse hybrid is viable. To solve the mechanism of hybrid inviability, sperm from one species was added to egg extracts from another species. After allowing replication to take place, metaphase extracts were added to create mitotic chromosomes. Staining single mitotic chromosomes with centromere markers revealed that roughly two paternal chromosomes completely lost CENP-A in the inviable hybrid cross, but not the viable cross (Gibeaux et al., 2018). Importantly, the loss of CENP-A could be rescued by addition of *X. laevis* CENP-A and HJURP (Kitaoka et al., 2022) (Figure 2F). Together these studies showcase the versatility of *Xenopus* egg extracts for studying the molecular underpinnings of chromatin assembly and compaction, which have important implications for speciation.

## Future perspectives: Bottom-up and spread out

Historically, *Xenopus* egg extracts have served as a powerful discovery platform for new mechanisms of genome remodeling. While many of the molecular players have now been discovered, the precise physical mechanisms are often unclear. Egg extracts are still the only cell-free system that allows for reconstitution of the entire nucleus, mitotic chromosomes, the centromere, and robust DNA replication, DNA repair, among other processes that were not described here. While depletion and add-back experiments are now possible in a few living systems, the biochemical manipulability of *Xenopus* egg extracts is unparalleled. Thus, reconstitution of chromatin using egg extracts will continue to serve a crucial role in the “messy middle” between pure *in vitro* reconstitution and *in vivo* studies. A major challenge in the chromatin field is bridging scales—how does nucleosome-level organization influence chromosome-scale or nuclear-scale activities? In the future, blending the newest approaches in biochemical reconstitution, imaging, and genomics will reveal new rules of genome organization and dynamics. For example, recent work reconstituting transcription in egg extracts combined with single particle cryo-EM has led to new mechanistic insights about DNA repair during transcription (Mevissen et al., 2024; Sato et al., 2025). While *Xenopus* excels as a biochemical and cell biological system for studying genome remodeling in the context of development, other systems are more efficient for performing genetic manipulations. Additionally, egg extracts are not ideal for studying transcription, as the genome not broadly activated until 5 hours post fertilization in the embryo (Jukam et al., 2017).

In addition to digging deeper into long-standing questions in the field, the future of the *Xenopus* system also lies in expanding the breadth of questions being asked. *Xenopus* species have evolved through a series of whole genome duplication events, resulting in a six-fold range in genome size (Miller et al., 2023). Recently, the species with the largest genome, the dodecaploid (12n) *Xenopus longipes* from Cameroon, has been introduced into the laboratory and is capable of laying eggs almost to the same extent as *X. laevis*, an allotetraploid (4n) (Loumont and Kobel, 1991; Miller et al., 2023). In the future it will be exciting to use *X. longipes* to answer long-standing evolutionary questions about the role of polyploidy in genetic innovation. Critically, the biochemical tractability of the *Xenopus* system will allow for discovery of new evolutionary strategies that allow genomes to adapt to major changes in size.
